# Regulation of Central Carbon and Amino Acid Metabolism in Plants

**DOI:** 10.3390/plants10030430

**Published:** 2021-02-24

**Authors:** Stefan Timm, Stéphanie Arrivault

**Affiliations:** 1 Plant Physiology Department, University of Rostock, Albert-Einstein-Straße 3, D-18051 Rostock, Germany; 2 Max Planck Institute of Molecular Plant Physiology, Wissenschaftspark Golm, Am Mühlenberg 1, D-14476 Potsdam-Golm, Germany; Arrivault@mpimp-golm.mpg.de

## 1. Introduction

Fluctuations in the prevailing environmental conditions, including light availability and intensity, CO_2_/O_2_ ratio, temperature, and nutrient or water supply, require rapid metabolic switches to maintain proper metabolism. To achieve this, a multitude of regulatory mechanisms are needed to communicate between the various metabolic branches to adjust fluxes through all routes related to central carbon and nitrogen metabolism. Despite considerable effort in understanding the genetics and biochemistry of plant primary metabolism, insights into the underlying mechanisms governing such acclimations and the involved regulatory circuits are still fragmentary. A better knowledge on those aspects would certainly help to engineer crop plants to maintain high yields under fluctuating environmental conditions.

Over the past decades, tremendous progress has been made to unravel the extraordinary complexity of plant primary metabolism from a biochemical and genetic point of view. Special attention has been paid to carbon and nitrogen assimilation and their subsequent metabolism [1,2,3]. Biochemical reactions of canonical pathways, such as the Calvin–Benson cycle, the oxidative pentose phosphate pathway, glycolysis, the tricarboxylic acid (TCA) cycle, and photorespiration have been thoroughly investigated [4,5,6,7,8]. However, given that many of those pathways run simultaneously, there are gaps regarding how these pathways are coordinated with each other, intertwined, and regulated. In this special issue, we invited authors to contribute to new studies encompassing the field of regulation of pathways that drive plant primary metabolism in oxygenic phototrophs, particularly focusing on photorespiration, photosynthesis, and glycolysis, as well as metabolite regulation, signaling and transport. In addition, natural acclimation strategies to a changing environment are included.

## 2. Photorespiration—A Key Driver for Adaptation to Molecular Oxygen and Abiotic Stresses

Photorespiration has attracted major interest in plant research over the past decades for different reasons. First, photorespiration is essential in all oxygenic phototrophs, as it degrades and recycles 2-phosphoglycolate (2-PG), which is formed in high amounts in the presence of oxygen during illumination through oxygenation of ribulose-1,5-bisphosphate via Rubisco [9]. Second, photorespiration has been identified as a key target to increase crop yield since the pathway itself releases CO_2_ during 2-PG recycling. Several strategies, such as synthetic bypasses or upregulation of enzyme activities, were successfully developed to manipulate photorespiratory flux, with promising outcomes in terms of stimulated photosynthetic carbon fixation [10,11]. Third, photorespiration is orchestrated in four subcellular compartments, including chloroplasts, peroxisomes, mitochondria, and the cytoplasm. Therefore, this pathway is an interesting example to study metabolite transport across membranes and interactions of the different subcellular compartments [12]. Fourth, the photorespiratory pathway also represents an example to study evolutionary aspects of photosynthetic metabolism [13]. Fifth, several pathway intermediates were shown to display a regulatory impact on the pathway itself or on other metabolic branches [11,14]. Hence, manipulation of the photorespiratory flux can be used for applied purposes and for basic research to shed more light on the communication between different metabolic branches under a changing environment [11].

In this issue, three studies tackle different aspects of research on photorespiration. Given the strong similarities of the cyanobacterial and plant photorespiratory pathways, it is assumed that photorespiration co-evolved with oxygenic photosynthesis in cyanobacteria and was endosymbiotically conveyed in eukaryotic algal lineages up to higher land plants [13]. This hypothesis was further confirmed by in silico analysis, but these data also revealed a dual origin of photorespiratory enzymes [15]. In particular, a strong controversy exists regarding the evolutionary origin of glycolate oxidase (GOX), predicted to originate from different sources [16]. However, in this issue, Kern and colleagues reanalyzed GOX evolution via a combination of phylogenetic and biochemical analyses using broad taxon sampling. These analyses strongly support the conclusion that GOX in higher plants evolved from a cyanobacterial ancestor protein [17]. A second study focused on the potential regulation of GOX activity though protein phosphorylation. Using site-directed mutagenesis and enzymatic measurements, Jossier et al. provided evidence that phosphorylation of GOX contributes to the regulation of enzymatic activities [18]. Finally, Timm et al. analyzed the potential for upregulation of photorespiratory enzymes to contribute to abiotic stress tolerance. These authors showed that faster degradation of 2-PG via increased phosphoglycolate phosphatase (PGLP) alleviated negative feedback of 2-PG on carbon-metabolizing reactions. In turn, photosynthesis was shown to be less O_2_-inhibited, indicating that it can operate more efficiently under unfavorable environmental conditions [19].

## 3. Maintenance of Growth and Photosynthesis under Fluctuating Conditions

In addition to evolutionary adaptations, the photosynthetic process also needs to react rapidly to various changes in the prevailing environmental conditions. In this issue, two research papers and one perspective paper dealing with these topics are presented. A key adaptation to declining CO_2_ concentrations during evolution was the establishment of inorganic carbon-concentrating mechanisms, including crassulacean acid metabolism (CAM) and C_4_ photosynthesis [20]. Since both CAM and C_4_ photosynthesis are present in *Portulaca grandiflora* leaves, and are simultaneously active, Guralnick et al. tested the hypothesis that both photosynthetic types are already developed and active in cotyledons [21]. Indeed, the authors provided evidence that both pathways are present and possibly run already at the early stages of leaf development. However, they also hypothesized that the CAM pathway is considerably slower compared to the C_4_ cycle [21]. The effects of short-term acclimation to changes in environmental conditions were analyzed using a collection of 36 randomly chosen Arabidopsis accessions. The study of Kaiser et al. demonstrated a large trait variation in growth and photosynthesis within this collection, especially under fluctuating conditions [22]. It seems likely to conclude that using such natural variation represents a valuable starting point to breed crop plants that are more robust under harsh environmental fluctuations. Finally, Walker et al. provided an interesting perspective on the flexibility of photosynthesis, in particular how the process can adapt and maintain high efficiency in a fluctuating environment [23].

## 4. Metabolite Regulation, Signaling, and Transport

One research paper and two review papers discuss different regulatory aspects in central carbon metabolism. Glycolysis is one of the major catabolic pathways for the breakdown of carbohydrates down to the TCA cycle to supply energy for different processes within the cell. A key enzyme of this pathway is pyruvate kinase, which transfers phosphate from phosphoenolpyruvate to ADP for ATP synthesis. In the study of Wulfert et al., five cytosolic pyruvate kinase isoforms were thoroughly studied. The authors provided strong evidence that these enzymes undergo several levels of regulation in order to adjust carbon flux through the glycolytic pathway. This includes differential transcription, allosteric metabolic regulation, and formation of subcomplexes among several isoforms [24]. With regard to metabolite regulation, Rosado-Souza et al. provided a comprehensive overview on the potential of ascorbate (vitamin C) and thiamine (vitamin B1) as metabolite signals. The authors summarized recent knowledge in the field and discussed that both molecules are of major importance for the communication between different metabolic branches in cellular organelles during acclimation processes [25]. Similarly, Toleco and colleagues focused on metabolite transport though membranes in cells of higher plants. In more detail, mitochondrial carriers were highlighted as gatekeepers, controlling carbon influx and efflux to regulate central carbon metabolism. A particular focus was on the interconnection of the cytoplasm with mitochondria. These compartments need to communicate and exchange metabolites to support the different flux modes of the TCA cycle, in particular through exchange of organic acids, or drive oxidative phosphorylation [26].

## 5. Conclusions and Outlook

The simultaneous operation and regulation of the different pathways involved in central carbon and nitrogen metabolism are highly complex. This special issue aims to provide a useful extension of the existing knowledge in the field and, hopefully, inspire new research to further develop this field including new strategies and projects. Such work would not only ultimately help to further increase our current understanding on how metabolism works and is regulated in different subcellular organelles, but will also contribute to the overall aim to breed better crops, showing high productivity in a changing environment.

## References

[1] Stitt M., Lunn J., Usadel B. (2010). Arabidopsis and primary photosynthetic metabolism—More than the icing on the cake. Plant J..

[2] Lawlor D.W. (2002). Carbon and nitrogen assimilation in relation to yield: Mechanisms are the key to understanding production systems. J. Exp. Botony.

[3] Nunes-Nesi A., Fernie A.R., Stitt M. (2010). Metabolic and Signaling Aspects Underpinning the Regulation of Plant Carbon Nitrogen Interactions. Mol. Plant.

[4] Raines C.A. (2003). The Calvin cycle revisited. Photosynth. Res..

[5] Nunes-Nesi A., Araújo W.L., Obata T., Fernie A.R. (2013). Regulation of the mitochondrial tricarboxylic acid cycle. Curr. Opin. Plant Biol..

[6] Plaxton W.C. (1996). The organization and regulation of plant glycolysis. Annu. Rev. Plant Biol..

[7] Nicholas J.K., von Schaewen A. (2003). The oxidative pentose phosphate pathway: Structure and organization. Curr. Opin. Plant Biol..

[8] Bauwe H., Hagemann M., Fernie A.R. (2010). Photorespiration: Players, partners and origin. Trends Plant Sci..

[9] Bauwe H., Hagemann M., Kern R., Timm S. (2012). Photorespiration has a dual origin and manifold links to central metabolism. Curr. Opin. Plant Biol..

[10] South P.F., Cavanagh A.P., Lopez-Calcagno P.E., Raines C.A., Ort D.R. (2018). Optimizing photorespiration for improved crop productivity. J. Integr. Plant Biol..

[11] Timm S., Hagemann M. (2020). Photorespiration—how is it regulated and how does it regulate overall plant metabolism?. J. Exp. Bot..

[12] Eisenhut M., Pick T.R., Bordych C., Weber A.P.M. (2012). Towards closing the remaining gaps in photorespiration—The essential but unexplored role of transport proteins. Plant Biol..

[13] Eisenhut M., Ruth W., Haimovich M., Bauwe H., Kaplan A., Hagemann M. (2008). The photorespiratory glycolate metabolism is essential for cyanobacteria and might have been conveyed endosymbiontically to plants. Proc. Natl. Acad. Sci. USA.

[14] Flügel F., Timm S., Arrivault S., Florian A., Stitt M., Fernie A.R., Bauwe H. (2017). The Photorespiratory Metabolite 2-Phosphoglycolate Regulates Photosynthesis and Starch Accumulation in Arabidopsis. Plant Cell.

[15] Kern R., Bauwe H., Hagemann M. (2011). Evolution of enzymes involved in the photorespiratory 2-phosphoglycolate cycle from cyanobacteria via algae toward plants. Photosynth. Res..

[16] Esser C., Kuhn A., Groth G., Lercher M.J., Maurino V.G. (2014). Plant and Animal Glycolate Oxidases Have a Common Eukaryotic Ancestor and Convergently Duplicated to Evolve Long-Chain 2-Hydroxy Acid Oxidases. Mol. Biol. Evol..

[17] Kern R., Facchinelli F., Delwiche C., Weber A.P.M., Bauwe H., Hagemann M. (2020). Evolution of Photorespiratory Glycolate Oxidase among Archaeplastida. Plants.

[18] Jossier M., Liu Y., Massot S., Hodges M. (2019). Enzymatic Properties of Recombinant Phospho-Mimetic Photorespiratory Glycolate Oxidases from Arabidopsis thaliana and Zea mays. Plants.

[19] Timm S., Woitschach F., Heise C., Hagemann M., Bauwe H. (2019). Faster Removal of 2-Phosphoglycolate through Photorespiration Improves Abiotic Stress Tolerance of Arabidopsis. Plants.

[20] Sage R.F. (2004). The evolution of C4photosynthesis. New Phytol..

[21] Guralnick L.J., Gilbert K.E., Denio D., Antico N. (2020). The Development of Crassulacean Acid Metabolism (CAM) Photosynthesis in Cotyledons of the C4 Species, Portulaca grandiflora (Portulacaceae). Plants.

[22] Kaiser E., Walther D., Armbruster U. (2020). Growth under Fluctuating Light Reveals Large Trait Variation in a Panel of Arabidopsis Accessions. Plants.

[23] Walker B.J., Kramer D.M., Fisher N., Fu X. (2020). Flexibility in the Energy Balancing Network of Photosynthesis Enables Safe Operation under Changing Environmental Conditions. Plants.

[24] Wulfert S., Schilasky S., Krueger S. (2020). Transcriptional and Biochemical Characterization of Cytosolic Pyruvate Kinases in Arabidopsis thaliana. Plants.

[25] Rosado-Souza L., Fernie A.R., Aarabi F. (2020). Ascorbate and Thiamin: Metabolic Modulators in Plant Acclimation Responses. Plants.

[26] Toleco M.R., Naake T., Zhang Y., Heazlewood J.L., Fernie A.R. (2020). Plant Mitochondrial Carriers: Molecular Gatekeepers That Help to Regulate Plant Central Carbon Metabolism. Plants.

